# The Accessible Vascular Indicators for Mild Cognitive Impairment Detection: The Predictive Value of the Ankle-Brachial Index

**DOI:** 10.3390/jcm14196991

**Published:** 2025-10-02

**Authors:** Agnieszka Gostyńska, Agata Puszcz, Nadia Kruszyńska, Marzena Bielas, Lucyna Woźnicka-Leśkiewicz, Anna Posadzy-Małaczyńska

**Affiliations:** 1 Faculty of Finance and Banking, Merito University of Poznań, 5 Powstańców Wielkopolskich Street, 61-895 Poznań, Poland; 2 Sexuology and Clinical Psychology Student Research Group, Department of Clinical Psychology, Poznań University of Medical Sciences, 70 Bukowska Street, 60-812 Poznań, Poland; 3 Department of Family Medicine, Poznań University of Medical Sciences, 49 Przybyszewskiego Street, 60-355 Poznań, Poland; n.kruszynska@ump.edu.pl (N.K.); mbielas@ump.edu.pl (M.B.); lucyna.woznicka@ump.edu.pl (L.W.-L.); annamalaczynska@ump.edu.pl (A.P.-M.)

**Keywords:** ankle-brachial index, neurocognitive disorders, dementia

## Abstract

**Objectives:** Neurocognitive disorders (NCDs) refer to a broad spectrum of conditions characterized by declining cognitive functions, such as memory, attention, language, and executive abilities. It is estimated that up to half of patients affected by NCDs remain undiagnosed or are diagnosed at an advanced stage of the disease. This study aimed to analyze the utility of subclinical organ damage markers, which could be used in primary care for the detection and prevention of NCD. **Methods**: The study participants (*n* = 137) completed neuropsychological tests (Addenbrooke’s Cognitive Examination/ACE and Mini-Mental State Examination/MMSE), a sociodemographic survey, an interview on past illnesses, and had their ankle-brachial index (ABI) and pulse wave velocity (PWV) values measured. **Results**: Based on the MMSE test, 26 participants (19.0%) were diagnosed with mild cognitive impairment (MCI) and 8 participants (5.8%) with NCDs. The study found that lower ABI values were associated with worse cognitive performance, suggesting that the ABI may be a useful tool for identifying individuals at increased risk of NCDs, while PWV cannot be used as a predictor for this group of diseases. **Conclusions**: Lower ABI values were associated with reduced cognitive performance, whereas PWV showed no significant relationship. The secondary findings suggest that physical activity, regular computer use, and better mental well-being were linked to improved cognitive outcomes. A low ABI value could potentially serve as a predictor of cognitive disorders, and as a diagnostic tool that is easily accessible and quick, it may improve diagnostics and the overall health of primary care patients. Health education regarding modifiable risk factors for dementia is also of crucial importance.

## 1. Introduction

In primary care practice, the growing number of patients with dementia syndromes presents an increasing challenge. According to WHO data, by 2030, the population of patients with dementia is expected to rise by approximately 50%, and by 2050, it could increase by as much as 250% [1]. Dementia syndromes also pose an economic problem, contributing annually to global losses at the level of two trillion US dollars [2]. Unfortunately, existing studies indicate that family doctors often overlook the assessment of cognitive function in their older patients. When cognitive function is assessed, the accuracy of diagnosing dementia is lower than 60%, and even lower for MCI [3].

MCI is a heterogeneous syndrome characterized by impairment in usually one cognitive domain. Researchers estimate that it affects around 10–30% of patients over the age of 60, with 10–20% progressing to Alzheimer’s disease, frontotemporal dementia, or dementia with Lewy bodies [4]. Patients commonly complain of forgetfulness regarding appointments, frequent misplacement of objects, problems with spatial orientation, or finding the right words; however, these are subjective complaints that do not impact daily behavior or independence. MCI can be diagnosed using objective methods, such as standardized tests—MMSE (Mini-Mental State Examination), ACE-III (Addenbrooke’s Cognitive Examination-III), or GPCOG (The General Practitioner Assessment of Cognition) when the test result is lower by 1.5 standard deviations from the expected value, and the criteria for neurocognitive disorders are not met [5].

According to neurologists and neuropsychologists, specialized diagnostic testing for neurocognitive disorders should be performed at the end of the diagnostic process, after excluding causes such as poisoning, alcohol or benzodiazepine abuse, delirium syndromes, mood disorders, or exacerbation of chronic disease [6]. This is a significant challenge for primary care physicians due to limited consultation time and the lack of many diagnostic tools [6]. Screening for dementia would be facilitated by an easily accessible biomarker or predictor, which numerous research teams have been searching for over the years.

Emerging evidence suggests that vascular health plays an important role in neurodegeneration. Lower ABI values may indicate peripheral arterial disease and impaired cerebral perfusion, while increased PWV reflects arterial stiffness, both of which have been implicated in cognitive decline [7,8,9,10]. Previous studies have shown that a low ABI, especially values below 0.9, is associated with cognitive impairment and dementia [11]. Among patients with lacunar infarction, a low ABI—but not elevated PWV—was independently linked to poorer MMSE performance [7]. Longitudinal data also indicate that a low ABI is predictive of worsening cognition over a 3-year period post-stroke [8]. Regarding arterial stiffness, recent systematic reviews have documented robust associations between higher PWV and cognitive decline in older adults, particularly those with hypertension [9]. Meta-analyses similarly demonstrate inverse relationships between aortic PWV and cognitive domains, such as memory and processing speed, and elevated levels of PWV increase the risk of cognitive impairment [10]. More broadly, arterial stiffness has been linked consistently to poorer cognition and small-vessel disease pathology [12].

Early identification of MCI enables timely lifestyle and pharmacological interventions, potentially delaying disease progression and improving patient quality of life. This study may serve as an important guideline in planning preventive and proactive measures. The conclusions from the study may help family doctors select the high-risk group for neurocognitive disorders and accelerate further diagnostics.

## 2. Materials and Methods

The research was conducted by Dr. Agnieszka Gostyńska as part of her doctoral dissertation preparation. This was a cross-sectional observational study conducted in a primary care setting. The study group (*n* = 137) consisted of conveniently sampled patients from consecutive Family Medicine clinics at the Department of Family Medicine, Karol Marcinkowski Medical University in Poznań. The project received approval from the Bioethics Committee at the Medical University of Poznań, resolution number 1061/16. No formal sample size calculation was performed due to the exploratory nature of the study.

**Figure 1:**
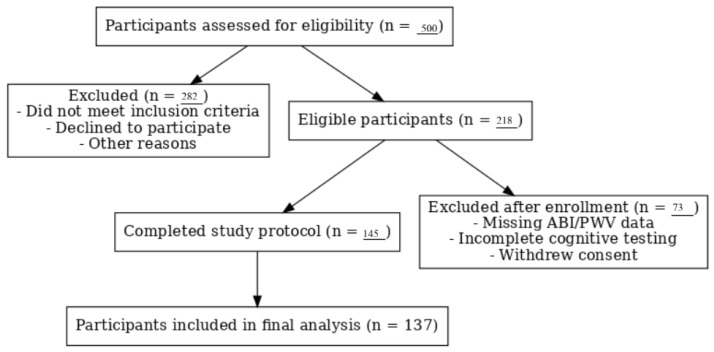
A flowchart of participant recruitment, exclusion, and inclusion. The figure illustrates the number of individuals assessed for eligibility, reasons for exclusion, and the final number of participants included in the analysis (*n* = 137).

Participants aged 50–76 years were intentionally recruited to capture individuals at elevated risk for cognitive impairment. The study period extended from January 2017 to December 2017, during which participant recruitment and data collection were conducted. Exclusion criteria included a history of unstable coronary artery disease, heart failure with an ejection fraction lower than 40%, cardiomyopathy, arrhythmias (not effectively treated with a pacemaker), heart defects, secondary hypertension, alcohol, drug or medication dependence, and stage 3 hypertension according to ESH guidelines. Other exclusion criteria included the presence of malignant tumors, hematological diseases, cirrhosis, kidney failure, neurological diseases, severe mental disorders, previously diagnosed dementia, and visual or auditory impairments preventing participation in the psychological assessments. A flowchart illustrating the number of participants assessed, excluded, and included in the final analysis is presented in Figure 1.

The study was divided into three stages. The first stage involved conducting an initial interview, obtaining written consent to participate, and gathering information regarding past illnesses, risk factors, and demographic and socioeconomic data. With patient consent, data from medical records were collected, including BMI, laboratory results, and relevant clinical history.

The second stage involved administering psychological tests: MMSE and ACE-III. MCI and NCDs were defined using MMSE thresholds similar to those reported in prior studies. A cutoff of ≤26 or MCI aligns with recommendations by Tsai et al. [13], who identified 27 as the optimal threshold for MCI (sensitivity 0.88, specificity 0.70), and ≤24 for dementia-level NCDs aligns with cutoffs associated with high positive predictive value (97%) in Alzheimer’s disease populations [14].

In the third stage, the ABI and PWV were measured using a BOSSO CardioVision ABPM-ABI device (BOSSO MedTech, Germany) validated for non-invasive vascular assessments [15].

The dependent variables included the following groups of variables: results of psychological tests (Table 1) and data from the interview regarding risk factors for cardiovascular disease (CVD) and NCDs. These data included past or current chronic diseases, family history of CVD, family history of NCDs, smoking, alcohol abuse, socioeconomic factors, history of CVD episodes, and medications taken. It is important to emphasize that risk factors for both CVD and NCDs are largely shared.

The independent variables included data from the BOSSO device (boso ABI-System 100 Bosch&Sohn): systolic and diastolic blood pressure, the ABI, and PWV. Additionally, medical records provided anthropometric data and laboratory results, including lipid profile and fasting glucose levels.

Sociodemographic variables were treated as secondary variables and included sex, age, education level, possession of a high school diploma, years of education, type of employment (manual or intellectual), and current and past occupational activity.

The study included 137 participants, comprising 73 women and 64 men, aged 50–76 years (M = 63.46 years, SD = 5.532). Nearly all participants (135 individuals) reported that they were currently not professionally active. Half of the participants had previously worked in manual labor (69 individuals), while the other half had worked in intellectual professions (68 individuals). In total, 21 participants had primary education, 37 had vocational education, 33 had secondary education, and 46 had higher education.

The study group was characterized using standard statistical descriptive methods: mean, standard deviation, percentage, minimum and maximum values, and their range. Statistical analysis was performed using SPSS 24 software. The variables were processed using the Mann–Whitney U test for non-parametric variables as data distribution was non-normal, Dunnett’s T3 test for variance analysis, and regression analysis, and the strength of associations between variables was described using Pearson’s correlation coefficient (r).

## 3. Results

Based on the formulated questionnaire, the socioeconomic conditions of the study group were assessed. The questions addressed both well-known risk factors for the development of dementia (such as depression, past trauma, bereavement, or loneliness) and the cognitive abilities of the participants. Only 27 participants reported living alone, the same number reported the loss of a close friend in the past year, and 20 individuals reported a lack of a close person. The vast majority (94.89%) stated that they did not have serious problems in their relationship with their spouse; three-quarters of the participants reported being able to share their emotions with loved ones; and 41.61% indicated that they had been exposed to traumatic events in the past. Among the participants, 25.55% reported a sense of hopelessness, 13.14% derived no pleasure from life, 16.79% experienced anxiety and distress, and 18.98% excessively worried. Dysphoric symptoms were slightly more frequent among the respondents than feelings of anxiety or depression. Almost 30% reported frequent anger outbursts over trivial matters, and 35% expressed excessive irritation due to the habits of others. A total of 57.66% of participants were satisfied with their income, but only 17.52% assessed their economic status as poor. Only half of the participants engaged in physical activity at least once a week. In total, 86.13% of respondents reported coping well with work demands. Additionally, 62.04% of participants used a home computer, which could be considered evidence of maintaining cognitive function or as a neuroprotective factor.

In the study group, 21.90% had experienced or were currently suffering from any mental disorder and 5.84% from any neurological disease, and 10.22% had a positive family history of dementia. Among the medications used in psychiatry and neurology, the largest group of respondents reported using sedative and hypnotic drugs (26.28%), followed by pro-cognitive medicines (16.06%) and antidepressants (13.87%). A smaller group reported using neuroleptics (10.95%) and anticonvulsants (5.84%).

**Figure 2:**
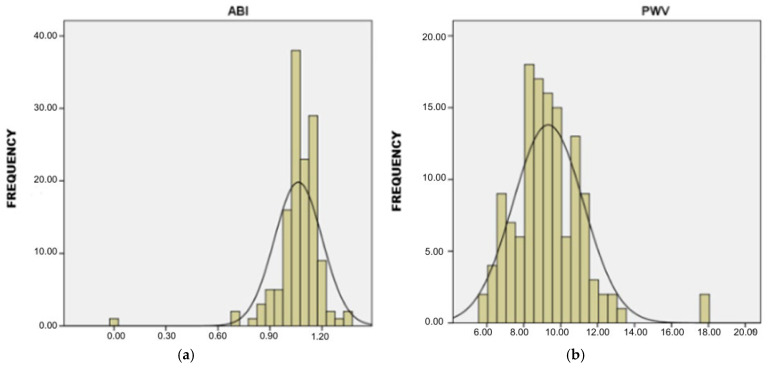
(**a**) The distribution of the ABI in the study sample. (**b**) The distribution of PWV in the study sample.

Using the BOSSO device, subclinical organ damage parameters were assessed: the ABI and PWV. The mean ABI in the study group was M = 1.064 (SD = 0.138), and the mean PWV was M = 9.320 m/s (SD = 1.908) (Figure 2a,b).

The cognitive abilities of the participants were assessed using standardized psychological tests. The average score on the MMSE test was M = 28.073 (SD = 5.11), indicating that most of the participants (*n* = 103) did not show cognitive impairments (Table 2). A score above 26 points on the MMSE test indicates no cognitive impairment, a score between 24 and 26 points suggests MCI, and a score below 24 points suggests probable dementia.

The average score on the ACE-III test was M = 86.037 (SD = 11.04), while the score on the abbreviated version, M-ACE, was M = 24.199 (SD = 4.788). The ACE-III test is more comprehensive than the MMSE, providing information on the entire profile of individual cognitive functions (Table 3) and demonstrating greater sensitivity in detecting dementia [16]. The maximum score on the test is 100; a score below 88 points suggests cognitive impairment with very high sensitivity, while a score below 82 points indicates cognitive impairment with very high specificity [17].

**Figure 3:**
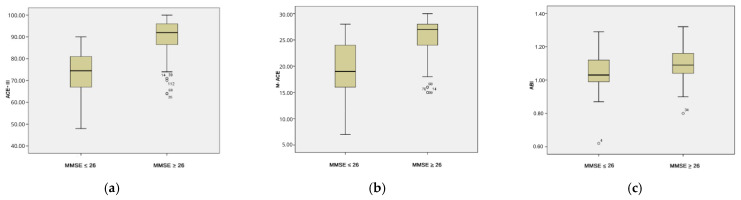
(**a**) The ACE-III score in individuals with NCDs (MMSE ≤ 26) and without NCDs (MMSE > 26). (**b**) The M-ACE subtest score for individuals with NCDs (MMSE ≤ 26) and without NCDs (MMSE > 26). (**c**) The ABI for individuals with NCDs (MMSE ≤ 26) and without NCDs (MMSE > 26).

The Mann–Whitney U test for non-parametric variables reveals statistically significant differences between the group with cognitive impairments (MMSE ≤ 26) and the group without cognitive impairments (MMSE > 26) in terms of ABI values (U = 1037.000; *p* = 0.005) and the results in the psychological tests ACE-III (U = 285.000; *p* = 25.882) and M-ACE (U = 579.000; *p* = 34.529). Participants with cognitive impairments identified in the MMSE test achieved lower scores in the ACE-III (Figure 3b) and M-ACE (Figure 3c) tests, as well as lower ABI values (Figure 3a) (Table 4).

The Pearson correlation coefficient (r) was calculated between the ACE-III (and M-ACE) test scores and subclinical organ damage measured by the ABI index, which was found to be statistically significant. Lower ACE-III and M-ACE scores were associated with lower ABI values (Table 5).

The relationship between cognitive functioning, assessed using the MMSE (Table 6) and ACE-III (Table 7) tests, and socioeconomic factors, such as computer usage, physical activity, traumatic experiences, and feelings of hopelessness, was examined using the Mann–Whitney U test. Individuals who achieved better results on the psychological tests reported that they regularly use a computer, engage in physical activity, and do not experience feelings of hopelessness. Interestingly, respondents with higher psychological test scores affirmed having experienced a traumatic event.

Factors such as receiving compensation proportional to effort, good work performance, not experiencing the loss of a close friend in the past year, and avoiding sharing personal thoughts with others were statistically significantly and positively correlated with high MMSE scores but did not affect ACE-III results. On the other hand, frequent irritation due to the habits of others showed a statistically significant positive correlation with high ACE-III scores but did not influence MMSE results.

**Figure 4:**
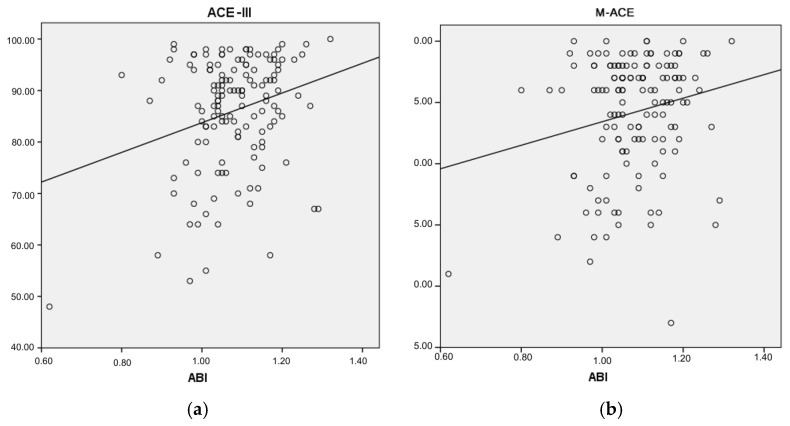
(**a**) The relationship between the dependent variable ACE III score and the predictor ABI; (**b**) the relationship between the dependent variable M-ACE score and the predictor ABI.

To assess the association between the ABI and cognitive performance, a univariate regression analysis was conducted. Linear models were generated for the relationship between the ACE-III scores and the ABI (Figure 4a), as well as for the relationship between the M-ACE scores and the ABI (Figure 4b).

The analyzed linear model (Figure 4a) was found to be a good fit for the data (F(1.135) = 9.668; *p* < 0.05). Based on the ABI values, 6.7% of the variance in the cognitive performance scores was explained by the association. The relationship is weak and positive (beta = 0.259). This means that a decrease of 0.1 in the ABI values was associated with 2.88 points in the ACE-III scores (B = 28.842).

The analyzed linear model (Figure 4b) was found to be a good fit for the data (F(1.135) = 5.531; *p* < 0.05). Based on the ABI values, 3.9% of the variance in the dependent variable can be predicted. The relationship is weak and positive (beta = 0.198). This means that a decrease of 0.1 in the ABI values was associated with 0.96 points in the M-ACE scores (B = 9.601).

## 4. Discussion

The purpose of the study was to assess the relationship between the occurrence of MCI and the coexistence of subclinical organ damage, measured by the ABI and PWV.

The ABI is a tool widely used in vascular surgery as a non-invasive method for assessing the degree of chronic ischemia in the lower limbs. It represents the ratio of the pressure at the ankle to the higher of the two brachial artery pressures. The calculated index value should fall within the range of 0.9–1.15. A lower value suggests narrowing of the lower limb arteries and a risk of ischemia, while a higher value indicates excessive vessel stiffness [18]. Many research teams have examined the usefulness of the ABI in monitoring chronic diseases other than chronic limb ischemia, such as coronary artery disease [19], diabetes [20], chronic kidney disease [21], and stroke [22].

The utility of the ABI in the context of cognitive disorders remains a topic requiring further exploration. In the APAC (Asymptomatic Polyvascular Abnormalities Community Study) involving over 3000 individuals, a correlation was found between impaired cognitive performance and a low ABI score [23]. Additionally, in the EPIDEMICA study, which involved a population of 1662 individuals over 65 years of age in Central Africa, the prevalence of cognitive disorders was 13.6%. This frequency was higher among individuals with an ABI ≤ 0.90 and an ABI ≥ 1.40 compared to those with ABI values within the range of 0.90 < ABI < 1.40 (20.1% and 17% vs. 12%, *p* = 0.0024) [24]. This study is particularly significant because approximately two-thirds of individuals with cognitive disorders live in developing countries, where access to advanced diagnostic tools is limited. Thus, the ABI, as an inexpensive and simple diagnostic tool, may serve as a valuable and effective solution for assessing the risk of cognitive disorders in this population.

In 2021, a study similar to the present one was conducted in Shanghai, where a research group with NCD symptoms (*n* = 217) and a control group without such symptoms (*n* = 259) were recruited. Regression analysis showed that a low ABI was an effective predictor of cognitive decline (*p* < 0.05). Pearson’s correlation analysis revealed that a low ABI (<0.9) had a significant impact on memory and spatial–visual functions within the cognitive domain (*p* < 0.05) [25]. In a systematic review by M. Guerchet et al. (2011) [11], 12 publications were analyzed, 6 of which conducted cross-sectional analyses, and 6 performed longitudinal analyses. All but one of the studies found a significant association between a low ABI and cognitive impairment, dementia, or Alzheimer’s disease.

In this study, a correlation was found between cognitive performance measured by the ACE-III test and subclinical organ damage measured by the ABI (Figure 4a). Regression analysis demonstrated that ABI values were significantly associated with cognitive performance scores in ACE-III and M-ACE (Figure 4a,b). Therefore, it can be inferred that lower ABI values were associated with poorer cognitive performance in our cohort; however, due to the cross-sectional design, these findings should not be interpreted as evidence of causality.

The second marker of subclinical organ damage analyzed in the study was PWV. The value of PWV depends on the stiffness, elasticity, and compliance of the arterial system [26]. In healthy vessels, which are elastic, the pulse wave propagates relatively slowly. As vessels become stiffer, the pulse wave propagates faster. High PWV thus suggests a higher risk for conditions such as hypertension, stroke, myocardial infarction, and presumably dementia. In the Sydney Memory and Ageing Study, after applying Bonferroni correction, no significant relationship between PWV and cognitive functions was found [27]. However, a separate analysis of this relationship in male and female groups showed that higher PWV values in men were associated with lower levels of overall cognitive function and memory. Nevertheless, no significant differences were observed in the relationship between PWV and cognitive functions between men and women. In the study by E. Nilsson et al. (2017) [28], PWV was measured in 3056 participants, followed by screening tests for neurocognitive performance. Patients who scored low were further diagnosed with dementia. A total of 159 cases of dementia were identified, including 57 chronic cases and 102 new cases. Logistic regression analysis showed that PWV was not associated with chronic dementia of any etiology.

In the present study, no association between PWV and lower cognitive performance was found; the relationship was not statistically significant in either the Mann–Whitney U test, Pearson correlation test, or regression analysis. This appears to be consistent with the results of studies conducted by other research teams.

Dean and Ayesha Sherzai developed their proprietary NEUROplan (Nutrition, Exercise, Unwind, Restore, Optimize), aimed at reducing modifiable risk factors for dementia [29]. Expanding on the acronym, the authors recommend following a Mediterranean or DASH diet, ensuring physical activity, training concentration and memory, introducing stress management techniques, promoting body regeneration, and optimizing cognitive reserve. The development, implementation, application, and evaluation of an individual plan for changing habits poses a significant challenge for both the patient and their primary care physician. However, the authors provide low-cost and appealing sets of cognitive and physical exercises, along with ready-to-use meal plans. The potential benefits of such preventive measures not only improve the health of the primary care physician’s patient population but also minimize the costs incurred by the state in the fight against dementia.

The study demonstrated a relationship between cognitive functioning, assessed both through the MMSE and ACE-III tests and physical activity participation. The socioeconomic interview conducted during the experiment included a question regarding whether the respondent engages in physical activity at least once a week for at least 30 min. Unfortunately, more than half of the participants (52.6%) answered negatively, even though 30 min of activity per week is considered significantly insufficient compared to the World Health Organization (WHO) recommendations, which suggest a minimum of 150 min of physical activity per week for older adults.

There is an increasing body of evidence that aerobic exercise improves cognitive functions [30]. In studies conducted by DeFina et al., involving nearly 20,000 patients from a preventive medicine clinic in Texas, it was shown that higher levels of physical activity in middle age reduce the risk of developing dementia later in life [31]. This relationship was observed both in individuals with and without a prior stroke, providing evidence that physical activity protects against dementia regardless of its cause, not just vascular dementia. The analysis by F. Sofi et al. (2010) [32], which included 15 prospective studies, demonstrated the neuroprotective role of physical activity, regardless of its intensity. The study sample consisted of 33,816 individuals without a dementia diagnosis, monitored throughout 1 to 12 years. During the observation period, a decline in cognitive functions was observed in 3210 participants. Statistical analysis, using a random effects model, showed that those engaging in intense physical activity were significantly protected against cognitive decline (−38%). Additionally, analysis of low and moderate levels of physical activity also revealed substantial protection against cognitive impairment (−35%). Healthcare professionals should encourage their patients to incorporate physical activity into their daily routine, not only due to its obvious benefits for cardiovascular health but also for its neuroprotective effects.

One of the most significant limitations of the study was the specific characteristics of the study group, which consisted of patients from a pre-clinical primary healthcare clinic. Due to the exploratory nature of the study, no control group was included, which limits the ability to compare the results with a healthy population. Each participant reported suffering from at least one chronic disease, which, on the one hand, introduces additional variables into the analysis, but on the other hand, is justified by the criteria for participant selection: the participants were patients who regularly visited their general practitioners. This group showed some homogeneity in terms of the types of past or existing illnesses, which can be attributed to the high prevalence of certain chronic diseases, such as hypertension or rheumatic diseases, among patients in such clinics.

Moreover, over 90% of the participants were regularly taking medications, yet the study did not account for either the potential side effects of these medications or their long-term impact on cognitive function. This is particularly relevant for medications such as benzodiazepines and statins, which are thought to have potential anti-cognitive effects. The study also did not consider the possible pro-cognitive and neuroprotective properties of nicotine, which represents another significant variable that could have influenced the study results [33].

Risk factors for various chronic diseases are often identical or tend to co-occur, making individuals suffering from one chronic condition particularly vulnerable to the development of other diseases. From a psychological and motivational perspective, the study participants were specific. First, participation in the experiment required participants to set aside free time, meaning that those with more free time were more likely to volunteer, including individuals not currently employed (72.3% of the participants). Second, some of the participants expressed concerns about their cognitive function, noticing subtle changes in their memory or having family histories of mental disorders, which might have influenced their motivation to participate in the study.

Data regarding the occurrence of neurodegenerative diseases in the participants’ families were obtained directly from the participants themselves. However, it should be noted that many of them lacked precise knowledge about the health of their parents or grandparents. It is important to emphasize that the participants’ parents and grandparents lived in the early 20th century, when knowledge about dementia and cognitive disorders was limited. Furthermore, a significant portion of the participants’ relatives died prematurely due to World War II, infectious diseases, or famine, without exhibiting cognitive disorders typical of old age.

Information on the participants’ socioeconomic conditions was obtained through interviews. Since all participants were aware that they were taking part in a study on risk factors for neurodegenerative diseases, there was a possibility of manipulating their responses to present themselves in a more favorable light. It is also worth noting that the study did not use standardized psychological questionnaires to assess depressive or dysphoric symptoms. Therefore, the actual mental state of the participants may have been difficult to assess objectively.

This study adds to the growing body of literature on vascular and cognitive health by simultaneously assessing the ABI, PWV, and key lifestyle-related factors within a primary care population. While previous studies have often examined these associations in hospital-based cohorts or highly selected research samples, our study focuses on patients attending routine family medicine clinics, reflecting a population more representative of real-world primary care. By integrating vascular measurements with cognitive screening and socioeconomic factors, this study provides a more comprehensive understanding of potential risk markers for early NCDs in everyday clinical settings.

This study has several limitations that should be considered when interpreting the findings. The cross-sectional design prevents establishing causal relationships between the ABI, PWV, and cognitive function, and the lack of a control group limits comparisons with the general population. The relatively small sample size and recruitment from a single primary care clinic may reduce generalizability and introduce selection bias. Although multiple risk factors were assessed, potential confounding variables, such as age, education, comorbidities, and medication use, were not fully adjusted for. Some information, including lifestyle habits and psychosocial factors, was self-reported and may be subject to recall bias. Finally, cognitive performance was assessed using screening tools (MMSE and ACE-III) rather than comprehensive neuropsychological testing, which may have led to misclassification in some cases. Larger, longitudinal studies are needed to confirm these findings and explore the potential role of the ABI as a screening tool in primary care.

Further research is needed to confirm our findings and clarify the clinical role of the ABI in cognitive assessment. Longitudinal studies in larger and more diverse populations are required to determine whether a low ABI precedes cognitive decline or simply reflects shared vascular risk factors. In addition, intervention trials could explore whether integrating ABI measurement into primary care screening helps identify high-risk individuals earlier and supports the implementation of targeted prevention strategies, such as lifestyle modification or vascular risk management. Combining the ABI with other biomarkers, neuroimaging, and comprehensive cognitive testing may further improve early detection and risk stratification for NCDs.

## 5. Conclusions

Lower ABI values were associated with poorer cognitive performance in this primary care cohort. The ABI, as a simple and widely available measure, may assist in identifying individuals at higher risk of MCI. Further longitudinal studies are required to establish causality. Patients who obtained lower scores in psychological cognitive function tests also had subclinical vascular organ damage.

Participants who engaged in regular physical activity achieved better results in cognitive function tests.

Participants who used computers more frequently, experienced fewer depressive symptoms, and had a past trauma experience, had a lower risk of developing NCDs.

Screening for subclinical organ damage in the population of family medicine patients can be a useful tool for the early diagnosis and prevention of NCDs.

## Figures and Tables

**Table 1:** Results of ACE-III and MMSE tests.

Variable	Measurement Tool	Indicator
Cognitive functioning—total score	ACE-III	Total score 0–100 points
Cognitive functioning—subscale	M-ACE	M-ACE score 0–30 points
Attention	ACE-III	0–18 points
Memory	ACE-III	0–26 points
Verbal fluency	ACE-III	0–14 points
Visuospatial functions	ACE-III	0–16 points
Language	ACE-III	0–26 points
Cognitive functioning	MMSE	0–30 points

**Table 2:** The assessment of neurocognitive impairment based on the MMSE test.

Category	*n*	%
NCD	8	5.839
MCI No Cognitive Impairment	26	18.978
	103	75.182

**Table 3:** The assessment of neurocognitive impairment in the ACE-III test.

ACE-III Scale	*n*	Mean	Max Score	Mean% Score	SD	Min–Max
Attention	137	16.759	18	93.007	1.468	12–18
Memory	137	21.139	26	81.482	4.793	6–26
Verbal Fluency	137	9.496	14	67.876	2.983	1–14
Language	137	24.051	26	92.182	2.712	9–26
Visuospatial Functions	137	14.511	16	90.941	1.728	9–16
**Total Score**	137	86.037	100	86.124	11.041	48–100
**M-ACE Score**	137	24.199	30	80.117	4.788	7–30

**Table 4:** Differences between participants with NCDs (MMSE ≤ 26) and without NCDs (MMSE > 26).

Variable	U Mann–Whitney	*p*-Value	Mean Rank—Cognitive Impairment	Mean Rank—No Cognitive Impairment
ABI Index	1037.000	0.005	48.000	75.932
ACE-III Total Score M-ACE Score	285.000	<0.001	25.882	83.233
	579.000	<0.001	34.529	80.379

**Table 5:** Neurocognitive functioning and subclinical organ damage.

	ABI	PWV
ACE-III Total Score	r = 0.259 **	NS
M-ACE	r = 0.198 *	NS

* *p* < 0.05, ** *p* < 0.01, NS—not statistically significant.

**Table 6:** The relationship between socioeconomic factors and MMSE scores.

Factor	Mann–Whitney U Value	*p*-Value	Yes	No
Computer use	911.000	0.000	84.282	44.019
Physical activity	1337.000	0.002	82.930	53.569
Difficulty coping with work demands	801.000	0.043	52.158	71.711
Salary disproportionate to the effort	1725.500	0.012	59.250	75.158
Loss of a close friend in the past year	1097.500	0.033	54.648	72.522
Feelings of hopelessness	1326.000	0.021	55.885	73.500
Avoiding sharing thoughts with others	1251.000	0.011	83.706	64.145
Past trauma	1710.500	0.011	78.991	61.881

**Table 7:** The relationship between socioeconomic factors and ACE-III scores.

Factor	Mann–Whitney U Value	*p*-Value	Yes	No
Computer use	515.000	0.000	88.941	36.404
Physical activity	1243.500	0.000	84.229	52.131
Feelings of hopelessness	1174.000	0.003	51.543	74.990
Irritation by other people	1690.500	0.044	78.281	63.994
Past trauma	1489.500	0.001	82.868	59.118

## Data Availability

The original data presented in the study are openly available in Zenodo at https://doi.org/10.5281/zenodo.14919033 (accessed on 24 Fubruary 2025).

## Abbreviations

The following abbreviations are used in this manuscript:

ABI	Ankle-Brachial Index
NCDs	Neurocognitive Disorders
PWV	Pulse Wave Velocity
CVD	Cardiovascular Disease
MCI	Mild Cognitive Impairment
ACE-III	Addenbrooke’s Cognitive Examination III
MMSE	Mini-Mental State Examination
